# Disparities in Healthcare and HBV Vaccination by Smoking Status: Findings from the National Health and Nutrition Examination Survey (NHANES) 2017–2018

**DOI:** 10.3390/healthcare12010041

**Published:** 2023-12-23

**Authors:** Wenxue Lin

**Affiliations:** Department of Epidemiology and Biostatistics, College of Public Health, Temple University, Philadelphia, PA 19122, USA; wenxue.lin@temple.edu

**Keywords:** smoking, healthcare, HBV, vaccination

## Abstract

Cigarette smokers face greater challenges in accessing healthcare compared with non-smokers. In the US, approximately 2.2 million individuals are chronically infected with hepatitis B virus (HBV). I used data from the National Health and Nutrition Examination Survey (NHANES) 2017–2018 to investigate the association between smoking status (current, former, and never smoker) and different health outcomes, including healthcare accessibility, HBV vaccination, general health condition, and health insurance. Multivariable logistic regressions were used to analyze healthcare disparity by smoking status. I found that current smokers had 40% higher odds (AOR = 1.4, 95% CI: 1.1, 1.8) of lacking routine healthcare access compared with non-smokers. Regardless of smoking status, I observed a high rate of HBV non-vaccination among all participants. Specifically, 64% of current smokers, 67% of former smokers, and 57% of non-smokers had not received a single dose of HBV immunization. My study sheds light on the persisting gaps in healthcare access, particularly for smokers, and the urgent need to promote awareness and vaccination against hepatitis B.

## 1. Introduction

Cigarette smoking is still the leading cause of preventable illness and death in the United States [1]. In 2021, 11.5% of U.S. adults (an estimated 28.3 million people) were current cigarette smokers [2]. The tobacco industry spends billions of dollars each year on cigarette advertising [2], including targeted marketing of menthol cigarettes to Black smokers [3,4]. Several studies suggest that menthol cigarette smokers may experience greater nicotine dependence, face more challenges in quitting [5], and demonstrate a reduced response to very low-nicotine-content (VLNC) cigarettes when compared with non-menthol cigarette users [6,7]. Carcinogenic chemicals found in cigarette smoke may interact with hepatitis B infection, resulting in a synergistic effect that damages liver cells and increases the risk of liver cancer [8]. Populations with low socioeconomic status and individuals with mental health issues have a higher prevalence of cigarette smoking compared with the general population [9,10,11]. Cigarette smokers may have limited access to healthcare services and encounter greater difficulties than non-smokers [12]. Healthcare access is crucial for smokers given their specific healthcare needs, potential elevated risk of lung carcinoma, and mental health issues [12]. However, limited evidence exists regarding disparities in healthcare access based on smoking status.

Hepatitis B virus (HBV) is primarily responsible for chronic liver disease, which contributes to liver cancer and cirrhosis [13,14,15]. According to the World Health Organization, more than 300 million people worldwide are living with chronic hepatitis B infection [13]. In the United States (US), an estimated 580,000 to 2.4 million people are affected by HBV [16,17]. HBV has become a significant public health concern, impacting both developing and developed countries. Although vaccination is the most effective way to prevent hepatitis [18], immunization rates (defined as the completion of a three-dose vaccine series) consistently remain low [19,20,21,22] because of factors such as a lack of knowledge and awareness of hepatitis B [18], financial issues [23], vaccine acceptance [24], failure to follow CDC guidelines, insufficient insurance coverage [25], and limited access to healthcare. For example, only 24.6% of adults report being vaccinated for HBV in the US [26], 33% of healthcare workers in Tanzania [21], and 23% of the general population of Korea [27].

In the US, enhancing healthcare access stands as a primary objective within the Healthy People 2030 framework [28]. The global hepatitis strategy of the World Health Organization (WHO) seeks to achieve a 90% reduction in new hepatitis infections and a 65% decrease in hepatitis-related deaths from 2016 to 2030 [29]. A comprehensive understanding of disparities in healthcare access and HBV vaccination based on cigarette smoking status may provide valuable insights regarding the Healthy People 2030 framework and the WHO’s hepatitis strategy. Therefore, my study aimed to assess the association between healthcare accessibility and smoking status (current, former, and non-smokers). Additionally, this study contrasted the differences in HBV vaccination among current, former, and non-smokers.

## 2. Materials and Methods

### 2.1. Study Design

For my current cross-sectional study, I used the National Health and Nutrition Examination Survey (NHANES) 2017–2018. The NHANES is a nationwide survey conducted by the National Center for Health Statistics (NCHS), which operates under the Centers for Disease Control and Prevention (CDC) [30]. The NHANES program commenced in the early 1960s and has since been conducted as a series of surveys, addressing various health-related subjects, such as nutrition, sexually transmitted diseases, anemia, diabetes, cardiovascular disease, environmental exposure, hearing impairment, oral health, and respiratory disease [30]. The NHANES interview process includes inquiries regarding demographics, socioeconomic status, dietary habits, and different aspects of health. The examination component consists of medical, dental, and physiological measurements, as well as laboratory tests [30]. These tests are conducted by exceptionally skilled medical professionals to maintain the program’s high standards of accuracy and reliability. The NHANES employs a complex, multistage, probability sampling design to assess the health and nutritional status of the US civilian, non-institutionalized population [30]. Information regarding survey questionnaires, study design, laboratory protocols, sampling weights, and the dataset is available on the CDC website (https://www.cdc.gov/nchs/nhanes/index.htm (accessed on 11 October 2023).

### 2.2. Participants

Out of the 9254 participants who completed the study, I excluded participants aged 18 years or younger (n = 3398), as well as those with missing data on outcomes (healthcare accessibility, HBV vaccination, general health condition, and health insurance) (n = 550), and covariates including age, sex (categorical, male vs. female), race/ethnicity (categorical, Hispanic/Mexican American vs. Non-Hispanic White vs. Non-Hispanic Black vs. all others), education (categorical, less than high school vs. high school or higher), body mass index (BMI), and ratio of family income to poverty (n = 1368). This resulted in a final sample of 3938; of these, 57.3% were non-smokers, 25.7% were former smokers, and 17% were current cigarette smokers.

### 2.3. Variables

I gathered information about participants’ smoking status from the NHANES 2017–2018 cigarette use survey. I classified individuals into three groups: current cigarette smokers, former cigarette smokers, and non-smokers, based on their responses to two survey questions: “Have you smoked at least 100 cigarettes in your entire life?” and “Do you now smoke a cigarette?” [31,32]. Current cigarette smokers were those who had smoked at least 100 cigarettes in their lifetimes and were currently using combustible cigarettes every day or on some days. Former cigarette smokers were defined as those who had smoked at least 100 cigarettes in their lifetimes but were not smoking cigarettes at the time of the survey. Non-smokers were participants who had never smoked or had smoked fewer than 100 cigarettes in their lifetimes [31,32].

I obtained sociodemographic variables, including age at screening (continuous), gender (categorical, male vs. female), race/ethnicity (categorical, Hispanic/Mexican American vs. Non-Hispanic White vs. Non-Hispanic Black vs. all others), education attainment (categorical, less than high school vs. high school or higher), body mass index (BMI, kg/m^2^), and ratio of family income to poverty (continuous) from demographics data in the NHANES 2017–2018. The response options for education attainment included “Less than 9th grade”, “9–11th grade (includes 12th grade with no diploma)”, “High school graduate/GED or equivalent”, “Some college or AA degree”, and “College graduate or above”. I dichotomized the response variables as “Less than high school (less than 9th grade and 9–11th grade, includes 12th grade with diploma)” vs. “High school or higher (high school graduate/GED or equivalent”, “Some college or AA degree”, and “College graduate or above)”. The ratio of family income to poverty is a measure of income in relation to a family’s financial requirements, calculated based on household income and government-defined poverty thresholds [33]. The ratio of family income to poverty serves as an indicator of the health and well-being of participants [34]. Previous research indicates a close relationship between the ratio of family income to poverty and children’s health, especially among those from low-income backgrounds in the United States [35]. Further, a higher ratio of family income to poverty is often associated with lower child morbidity rates [35].

To assess the association between healthcare accessibility and smoking status (current, former, and non-smokers), I used the following access-to-care survey question: “Is there a place that you usually go when you are sick or you need advice about your health?” The response options for routine healthcare access included “Yes”, “There is no place”, and “There is more than one place”. I categorized the response variables as “Yes or there is more than one place” vs. “There is no place” for analysis. I used an immunization questionnaire (“Have you ever received the 3-dose series of the hepatitis B vaccine?”) to make a comparison of HBV vaccination rates among current, former, and non-smokers. Response options to HBV vaccination included “Yes, at least 3 doses”, “less than 3 doses”, and “No doses”. I collapsed the participants’ responses into a binary variable (“Yes, at least 3 doses or less than 3 doses” vs. “No doses”) for ease of comparison and interpretation.

In addition to the primary aim (access to a healthcare place) and the secondary aim (HBV vaccination), I included general health condition (“Would you say your health in general is”) and health insurance (“Are you covered by health insurance or some other kind of healthcare plan?”) as study outcomes. Responses regarding general health condition were categorized as “Excellent, very good, good“ vs. “Fair or poor”, and health insurance responses included “Yes” vs. “No”. I excluded “Refused”, “Don’t know”, and missing responses from my analysis. There were four dependent outcomes: (1) access to a healthcare place, (2) HBV vaccination, (3) general health condition, and (4) health insurance status. There was one main independent variable (exposure), which was smoking status, categorized as current smokers, former smokers, and non-smokers.

### 2.4. Statistical Analysis

I analyzed the relationship between categorical and continuous descriptive variables using the Rao–Scott χ^2^ tests and Analysis of Variance (ANOVA) tests, respectively [32,36]. Weighted multivariable logistic regression models were employed to assess the relationship between smoking status (current, former, and never smoker) and different outcomes (healthcare accessibility, HBV vaccination, general health condition, and health insurance), while controlling for demographic characteristics, including gender, race/ethnicity, age, education, BMI, and the ratio of family income to poverty [37]. Model 1 shows the crude model with no adjustments for confounders, and Model 2 is adjusted for the sociodemographic confounders mentioned earlier.

For all statistical analyses, I used SAS statistical software version 9.4 (SAS Institute Inc., Cary, NC, USA) with a two-sided significance level of 0.05. SAS SURVEY Procedures, including PROC SURVEYMEANS, PROC SURVEYFREQ, and PROC SURVEYLOGISTIC, were employed for statistical analysis, incorporating appropriate weights, strata, and clustering variables to account for the complex sampling design of the NHANES [37,38,39].

## 3. Results

Table 1 presents the sample characteristics based on smoking status using NHANES data from 2017 to 2018. Of all eligible participants, 57.3% were non-smokers, 25.7% were former smokers, and 17% were current cigarette smokers. My study consisted of 47.7% males and 52.3% females with an average age of 49.4. Current smokers were more likely to be younger (45.3 vs. 48.4), be male (52.6% vs. 40.2%), be Non-Hispanic White (65.2% vs. 61.8%), be less educated (15.6% vs. 7.8%), have no routine healthcare access (27.9% vs. 16.4%), receive no doses for HBV vaccination (64.3% vs. 57.3%), self-identify as being in fair or poor health (27.8% vs. 14.7%), lack health insurance coverage (21.4% vs. 9.8%), have a lower BMI (28.7 vs. 29.7), and have a lower ratio of family income to poverty (2.2 vs. 3.3) compared with non-smokers. Former smokers were more likely to be older (54.6 vs. 48.4), be male (61.3% vs. 40.2%), be Non-Hispanic White (72.5% vs. 61.8%), be less educated (11.5% vs. 7.8%), receive no doses for HBV vaccination (67.1% vs. 57.3%), self-identify as being in fair or poor health (18.9% vs. 14.7%), and have a higher BMI (31.0 vs. 29.7) than to non-smokers.

The association between smoking status and healthcare access, HBV vaccination, general health condition, and health insurance is presented in Table 2. In the crude model (Model 1), current smokers had 90% higher odds of lacking routine healthcare access, 30% higher odds of not receiving HBV vaccination, 120% higher odds of reporting fair or poor health, and 150% higher odds of being uninsured compared with non-smokers. After adjusting for confounders (Model 2), current smokers still exhibited 40% higher odds of lacking routine healthcare access compared with non-smokers (AOR = 1.4, 95% CI: 1.1, 1.8). Further, I also observed a significant association between smoking status and HBV vaccination. Specifically, current smokers had 50% higher odds of not receiving the hepatitis B vaccine compared with non-smokers (AOR:1.5, 95% CI: 1.1, 1.9) after adjusting for gender, race/ethnicity, age, education, BMI, and the ratio of family income to poverty. Further, current smokers had twice the odds of self-identifying as being in fair or poor health than non-smokers (AOR = 2.0, 95% CI: 1.5, 2.7). There was no significant difference in healthcare access, HBV vaccination, general health condition, or insurance status between former smokers and non-smokers with or without adjusting for confounders (Table 2; Models 1 and 2).

## 4. Discussion

My study revealed that over 27% of current smokers lack a routine place to go for healthcare and rate themselves as being in fair or poor health, and approximately 21% of them lack health insurance or access to government healthcare programs (such as Medicare and Medicaid). Additionally, more than 57% of participants did not receive any doses of the HBV vaccination, regardless of their smoking status.

My research findings align with previous studies that have emphasized a lack of a usual source of care (USC) for smokers. USCs play an important role in providing essential health services to individuals with smoking-related illnesses or seeking health consultations [12,40,41]. It is widely acknowledged that smoking leads to a range of adverse health effects, such as chronic obstructive pulmonary disease, heart disease, stroke, lung cancer, diabetes, and various respiratory diseases, arising from the carcinogenic compounds found in combustible cigarette smoke [1]. The disproportionate prevalence of smoking among vulnerable populations, including those with mental health concerns [10,11] and individuals with low socioeconomic status [42], underscores a significant public health concern. These vulnerable cigarette users, already burdened by health disparities [37], are even more susceptible to the detrimental effects of smoking given their limited access to healthcare services. As a result, it becomes imperative for smokers to have access to healthcare resources [12]. However, smokers encounter a series of barriers that hinder their ability to access healthcare, especially when compared with non-smokers [12]. For instance, the existing challenges can be attributed to a combination of unmet healthcare needs, the absence of a reliable USC [12], and a lack of a regular healthcare place for smokers, as evidenced by my study, where current smokers had 40% higher odds of not having a routine place to go for healthcare compared with non-smokers.

Regardless of smoking status, I observed a low HBV vaccination rate among all participants. Specifically, 64% of current smokers, 67% of former smokers, and 57% of non-smokers had not received a single dose of HBV immunization. In the US, approximately 2.2 million individuals are chronically infected with hepatitis B [43], contributing to over 2000 deaths annually [44]. HBV-infected patients often remain asymptomatic, with only 10% of hepatitis B patients being aware of their disease [45]. Further, patients may unknowingly transmit this infection through various means, including perinatal transmission (from mother to child at birth), needlestick injuries, tattooing, piercing, direct contact with infected blood or body fluids [45], unprotected sexual intercourse, and the use of medical and dental equipment contaminated with HBV [46,47]. Patients living with untreated chronic hepatitis B face a higher risk of developing end-stage liver diseases such as liver failure, cirrhosis, and hepatocellular carcinoma [43,48,49,50,51]. More than half of hepatocellular carcinoma cases are attributable to HBV infection in the world [52].

The most effective way to prevent hepatitis B virus (HBV) infection is through vaccination [53]. HBV vaccines have proven highly effective when administered in the recommended three-dose schedules [46]. However, according to a US National Health Interview Survey (NHIS) study on vaccination coverage, only 24.6% of adults aged 19 and older had been vaccinated, along with 16.5% of adults aged 50 and older. High-risk adults, as defined by the US CDC, which includes individuals with sex partners with hepatitis B; men who have sexual contact with other men; and people who share needles, syringes, or other drug-injection equipment, etc., have a vaccination rate of less than 50% [26,54]. Of particular concern is the finding that almost more than two-thirds of current and former cigarette smokers had not received any HBV vaccination, with current smokers having 50% higher odds of not receiving HBV vaccination compared with non-smokers. This is worrisome since the carcinogenic chemicals found in cigarette smoke can potentially interact with hepatitis B infection, leading to a synergistic effect that damages liver cells and increases the risk of liver cancer [8].

Several states have expanded Medicaid coverage to low-income adults aged 19 to 64 because of the Affordable Care Act (ACA). Consequently, the proportion of uninsured adults decreased from 15% to 7.8% between 2013 and 2017, demonstrating the positive impact of Medicaid coverage expansion [55]. In my study, I found that approximately 9% of former smokers and non-smokers were uninsured. However, more than 21% of current smokers remained without insurance or access to Medicaid/Medicare. Similarly, Teferra et al. revealed that the most significant improvements in healthcare access could be observed among never smokers, but there was no noticeable change or progress among current smokers when they assessed the impact of Medicaid expansion in Ohio [12]. It is possible that smokers’ knowledge and risk perceptions regarding smoking [56,57] and lower health insurance literacy [58] may contribute to the less pronounced effect of Medicaid expansion on smokers’ healthcare access and insurance status [12].

The association between socioeconomic status and smoking addiction is significant. For instance, lower levels of education are associated with higher smoking rates, and correspondingly, lower education levels are linked to increased probabilities of tobacco-related cancers [59]. Further, there exists an inverse relationship between the level of education attained and premature mortality rates among adults [59]. Thus, socioeconomic factors, such as educational attainment, play important roles in smoking behavior and the occurrence of tobacco-related diseases and mortality.

I found discrepancies in healthcare accessibility, HBV vaccination rates, and health insurance coverage based on gender (Supplement Table S1). Specifically, men had higher odds than women of lacking a place for healthcare (AOR = 2.4), not receiving a single dose of HBV vaccine (AOR = 1.6), and being uninsured (AOR = 1.8) (Supplement Table S1). Additionally, previous studies have suggested that the health status of unemployed individuals is worse than those who are employed [60]. Consistent with this finding, my research indicated that individuals below the federal poverty level have significantly higher odds of lacking healthcare access (AOR = 1.5), reporting fair or poor health (AOR = 2.4), and lacking health insurance coverage (AOR = 2.8) compared with those above the federal poverty level (Supplement Table S1).

Quitting smoking is one of the most effective means of enhancing the health of cigarette smokers, regardless of age or the duration of smoking [61]. For instance, quitting smoking can lead to an extension of life expectancy by up to a decade and a substantial reduction in the risk of premature mortality, cardiovascular disease, strokes, markers of inflammation and hypercoagulability, coronary heart disease, and adverse reproductive health outcomes [61]. Further, the act of quitting smoking significantly reduces the risk of developing 12 types of cancer, including bladder cancer, lung cancer, kidney cancer, liver cancer, oral cavity and pharynx cancer, and colon and rectum cancer, among others [61]. This reduction in cancer risk underscores the positive impact of quitting on smokers’ health. In my study, I found that, while current smokers exhibit significantly higher odds of lacking routine healthcare access compared with non-smokers, a similar association does not extend to former smokers. In addition, former smokers, after quitting, report general health statuses that closely mirror those of non-smokers, as well as similar insurance statuses. Former smokers not only evade the healthcare disparities faced by current smokers but also align with non-smokers in terms of healthcare access, overall health condition, and insurance coverage. In summary, quitting smoking offers an extended lifespan, a reduced risk of life-threatening diseases, and similar overall health outcomes and healthcare accessibility.

In 2018, the U.S. Food and Drug Administration (FDA) issued an Advanced Notice of Proposed Rulemaking (ANPRM) aimed at reducing nicotine in tobacco products [60]. Subsequently, in 2022, the FDA proposed product standards to prohibit menthol as a characterizing flavor in cigarettes [62]. These efforts hold the potential to yield significant public health benefits, such as preventing 16 million people from smoking by 2060 and avoiding approximately 8.5 million tobacco-related deaths in the United States by 2100 [63]. Very low-nicotine-content (VLNC) cigarettes, with nicotine levels of less than 0.2 mg of nicotine per cigarette or 0.4 mg of nicotine per gram of tobacco, have been extensively studied regarding their treatment effects [64,65]. Several randomized controlled trials have highlighted the benefits of VLNC, including reduced smoking and tobacco toxicant exposure, a decrease in the number of cigarettes smoked per day, an increase in quitting attempts, and lower dependence compared with usual-nicotine-content cigarette users [66,67,68,69,70,71,72]. Two common methods for VLNC cigarettes are immediate reduction, which rapidly lowers nicotine content from the standard 11.6 mg per cigarette to VLNC levels [70,72], and gradual reduction, where nicotine content is progressively reduced through multiple stages, ranging from 11.6 mg of nicotine per cigarette to 7.4, 3.3, 1.4, 0.7, and 0.2 mg of nicotine per cigarette [64,65]. However, a potential concern regarding VLNC cigarettes is their acceptability, compliance, and subjective responses. Some participants have reported reduced satisfaction with VLNC cigarettes compared with usual-nicotine-content cigarettes, primarily because of the lower nicotine content [64,73]. In addition to VLNC, the FDA has taken further steps to ban menthol in cigarettes, a flavor that is heavily promoted by tobacco industries and is particularly popular among African Americans [74]. An estimated 85% of African American smokers use menthol cigarettes [75]. A simulation model, based on data from the National Health Interview Survey (NHIS), has shown that menthol cigarettes contributed to over 10 million additional smokers, 3 million lost life years, and at least 350,000 premature deaths from 1980 to 2018 [76]. Further, the presence of menthol in VLNC cigarettes may negatively affect their treatment effect, as observed in studies where the reduction in smoking toxicant exposure was smaller in menthol VLNC cigarette users compared with non-menthol VLNC users [6]. Another study indicated that menthol may also negatively impact study adherence, with non-menthol smokers demonstrating better adherence to VLNC cigarettes compared with menthol cigarette users [7]. While the FDA and public health agencies have made significant strides in reducing the prevalence of cigarette smoking and the harmful effects caused by it, implementing VLNC cigarettes and banning menthol still poses various challenges that warrant public attention.

My study has several limitations. Firstly, the NHANES study population consists of the US civilian, non-institutionalized population, whereas institutionalized populations typically exhibit a higher prevalence of HBV infection [77]. Therefore, the association I observed between HBV vaccination and smoking status may not be applicable to other countries [78] or institutionalized people. Secondly, the former smokers with varying durations since quitting might have disparities in healthcare utilization [37]. However, I did not further classify former smokers based on the years since quitting (e.g., <2 years, 2–4 years, or 5–9 years). Thirdly, my study analyzed combustible cigarette smokers only, but it is worth noting that the prevalence of electronic cigarettes (e-cigarettes) continues to rise in the United States [79]. The dual use of combustible cigarettes and e-cigarettes raises additional concerns for public health, given the ongoing debate over the long-term health effects of e-cigarettes [80,81,82,83]. Further, the knowledge and risk perceptions of cigarette smoking [84,85] and HBV may differ based on the use of different tobacco products. One of the future research directions could be assessing healthcare access, knowledge, risk perceptions [86] related to smoking, and vaccination rates among individuals who engage in the dual use of combustible cigarettes and e-cigarettes. This examination could provide important insights into the healthcare access of the dual-use population, offering potential targeted public health interventions. My study excluded participants under the age of 18. Therefore, another research direction could be examining healthcare access and vaccination rates among youth based on smoking status. This is particularly important given the substantial concerns surrounding vaping among young individuals, with 2.55 million US middle and high school students reporting current e-cigarette use in 2022 [87]. Another limitation of my study could be attributed to the missing data on smoking status and outcomes, such as healthcare accessibility, HBV vaccination, general health condition, and health insurance. The missing data could diminish the statistical power and potentially lead to biased estimates in the study [88]. Further, my study did not control for healthy vaccine bias, which could narrow the difference in vaccination rates by smoking status [89]. In addition to smoking status, examining other variables such as risk perceptions and knowledge of vaccination available in NHANES data could justify the disparities in access to healthcare and specific HBV vaccination, offering potential directions for future study and investigation. I used a cross-sectional study design, and the limitation is the uncertainty of temporality. Future research may consider using multiple cycles of data from the NHANES and enhance the study design by conducting a longitudinal study.

Overall, my study sheds light on the persisting gaps in healthcare access, especially for smokers, and the urgent need to promote awareness and vaccination against hepatitis B. Addressing these issues is crucial to improving healthcare access and reducing disparities, not only for smokers but also for vulnerable populations who use cigarettes.

## 5. Conclusions

The findings from my study emphasize the concerning healthcare disparities faced by cigarette smokers. The low rates of HBV vaccination across diverse populations, especially among smokers, underscore the urgent need for widespread immunization. Addressing disparities in healthcare access and promoting vaccination not only prevent the spread of hepatitis B but also alleviate the associated risks of liver diseases and liver cancer.

## Figures and Tables

**Table 1:** Characteristics of participants by smoking status.

	Current Smokers N = 717 (17%)	Former Smokers N = 980 (25.7%)	Non-Smokers N = 2241 (57.3%)	Total N = 3938	*p*-Value
Gender					**<0.001**
Male	415 (52.6)	600 (61.3)	869 (40.2)	1884 (47.7)	
Female	302 (47.3)	380 (38.7)	1372 (59.8)	2054 (52.3)	
Race/ethnicity					**<0.001**
Hispanic	90 (10.1)	196 (12.0)	508 (15.0)	794 (13.4)	
NH-White	314 (65.2)	459 (72.5)	690 (61.8)	1463 (65.1)	
NH-Black	206 (13.5)	194 (7.1)	530 (12.3)	930 (11.2)	
Others	107 (11.2)	131(8.4)	513 (10.9)	751 (10.3)	
Education					**<0.001**
<High school	162 (15.6)	201 (11.5)	350 (7.8)	713 (10.1)	
≥High School	555 (84.3)	779 (88.5)	1891 (92.2)	3225 (89.9)	
Place to go for healthcare					**<0.001**
At least one place	530 (72.1)	869 (87.3)	1850 (83.6)	3249 (82.6)	
No place	187 (27.9)	111 (12.7)	391 (16.4)	689 (17.4)	
HBV vaccination					**0.02**
At least one doses	220 (35.7)	245 (32.9)	854 (42.7)	1319 (39.0)	
No doses	497 (64.3)	735 (67.1)	1387 (57.3)	2619 (61.0)	
General health condition					**<0.001**
Excellent, very good, or good	476 (72.2)	713 (81.1)	1798 (85.3)	2987 (82.0)	
Fair or poor	241 (27.8)	267 (18.9)	443 (14.7)	951 (18.0)	
Covered by health insurance					**<0.001**
Yes	559 (78.6)	887 (90.7)	1969 (90.2)	3415 (88.3)	
No	158 (21.4)	93 (9.3)	272 (9.8)	523 (11.7)	
Age, year	45.3 (0.9)	54.6 (1.1)	48.4 (0.66)	49.4 (0.6)	**<0.001**
BMI (kg/m^2^)	28.7 (0.4)	31.0 (0.4)	29.7 (0.2)	29.8 (0.2)	**<0.001**
Ratio of family income ^1^	2.2 (0.1)	3.3 (0.07)	3.3 (0.06)	3.1 (0.1)	**<0.001**

Data source: NHANES 2017–2018. ^1^ Ratio of family income to poverty. Categorical variables: % (standard error). Continuous variables: mean (standard error). *p*-values were calculated with the Rao–Scott x^2^ test and ANOVA for categorical variables and continuous variables, respectively. Bold *p*-values indicate significance.

**Table 2:** The association between smoking status and healthcare accessibility, HBV vaccination, general health condition, and health insurance status.

	Non-Smoker OR (95% CI)	Former Smoker OR (95% CI)	Current Smoker OR (95% CI)	*p*-Value
Model 1 (Crude)				
Place to go for healthcare				**<0.001**
No place	1.00 (Reference)	0.7 (0.5, 1.0)	1.9 (1.6, 2.4)	
HBV vaccination				**0.02**
No doses	1.00 (Reference)	1.5 (1.0, 2.3)	1.3 (1.1, 1.6)	
General health condition				**<0.001**
Fair or poor	1.00 (Reference)	1.4 (1.0, 1.7)	2.2 (1.7, 2.9)	
Covered by health insurance				**<0.001**
No	1.00 (Reference)	0.9 (0.6, 1.6)	2.5 (1.7, 3.7)	
Model 2 (Adjusted) ^1^				
Place to go for healthcare				**0.02**
No place	1.00 (Reference)	0.8 (0.6, 1.2)	1.4 (1.1, 1.8)	
HBV vaccination				**0.04**
No doses	1.00 (Reference)	1.1 (0.7, 1.6)	1.5 (1.1, 1.9)	
General health condition				**<0.001**
Fair or poor	1.00 (Reference)	1.0 (0.8, 1.4)	2.0 (1.5, 2.7)	
Covered by health insurance				0.09
No	1.00 (Reference)	1.1 (0.6, 2.1)	1.5 (1.0, 2.4)	

^1^ Adjusted for demographic characteristics, including gender, race/ethnicity, age, education, BMI, and the ratio of family income to poverty. Models the odds of having no place to go for healthcare, having no HBV vaccination, being in fair or poor health condition, and not being covered by health insurance. Bold *p*-values indicate significance.

## Data Availability

The NHANES data are publicly available on the CDC website: https://www.cdc.gov/nchs/nhanes/index.htm (accessed on 18 August 2023).

## Supplementary Materials

The following supporting information can be downloaded at https://www.mdpi.com/article/10.3390/healthcare12010041/s1: Table S1: The association between gender and socioeconomic status and healthcare accessibility, HBV vaccination, general health condition, and health insurance status.
