# Supplementary material for: Disparities in Healthcare and HBV Vaccination by Smoking Status: Findings from the National Health and Nutrition Examination Survey (NHANES) 2017–2018
Source: Healthcare (Basel). 2023 Dec 23;12(1):41. doi: 10.3390/healthcare12010041 (PMC10779267; doi:10.3390/healthcare12010041)
Supplement: Supplementary file 1 [file healthcare-12-00041-s001.zip › healthcare-2725400-supplementary.pdf]

Table S1. The association between gender and socio-economic status and healthcare accessibility, HBV vaccination, general health condition, and health insurance status.

	Female OR (95% CI)	Male OR (95% CI)	p-value
Model 2 (Adjusted)[1]			
Place to go for healthcare			**<0.001**
No place	1.00 [Reference]	2.4(1.9,3.0)	
HBV vaccination			**0.0003**
No doses	1.00 [Reference]	1.6(1.3,2.0)	
General health condition			0.5
Fair or poor	1.00 [Reference]	0.9(0.8,1.1)	
Covered by health insurance			**0.0003**
No	1.00 [Reference]	1.8(1.4,2.3)	
	Federal ≥ 100%[3] OR (95% CI)	Federal < 100% OR (95% CI)	
Model 2 (Adjusted)[2]			
Place to go for healthcare			**0.03**
No place	1.00 [Reference]	1.5(1.0,2.0)	
HBV vaccination			0.93
No doses	1.00 [Reference]	1.0(0.7,1.5)	
General health condition			**<0.001**
Fair or poor	1.00 [Reference]	2.4(1.7,3.3)	
Covered by health insurance			**<0.001**
No	1.00 [Reference]	2.8(2.0,4.0)	

[1]Adjusted for demographic characteristics, including race/ethnicity, age, education, BMI, smoking status, and the ratio of family income to poverty. [2]Adjusted for demographic characteristics, including gender, race/ethnicity, age, education, smoking status, and BMI. [3]Federal poverty level. Modeled the odds of no place to go for healthcare, no HBV vaccination, fair or poor health condition, and not covered by health insurance. Bolded p-value indicated significance.
